# Assessing the construct validity of the Quality-of-Life-Aged Care Consumers (QOL-ACC): an aged care-specific quality-of-life measure

**DOI:** 10.1007/s11136-022-03142-x

**Published:** 2022-06-09

**Authors:** J. Khadka, J. Ratcliffe, C. Hutchinson, J. Cleland, B. Mulhern, E. Lancsar, R. Milte

**Affiliations:** 1 Health and Social Care Economics Group, Caring Future Institute, College of Nursing and Health Sciences, Flinders University, Sturt North, GPO Box 2100, Adelaide, SA 5001 Australia; 2 Registry of Senior Australians, South Australian Health and Medical Research Institute, Adelaide, Australia; 3 Centre for Health Economics Research and Evaluation, University of Technology Sydney, Sydney, NSW Australia; 4 Department of Health Services Research and Policy, Australian National University, Canberra, ACT Australia

**Keywords:** Quality of Life, Preference-based measure, Residential-aged care, Community-aged care

## Abstract

**Purpose:**

To evaluate the construct (convergent and known group) validity of the Quality-of-Life-Aged Care Consumer (QOL-ACC), an older-person-specific quality-of-life measure designed for application in quality assessment and economic evaluation in aged care.

**Methods:**

Convergent validity was assessed by examining relationships with other validated preference-based measures (EQ-5D-5L, ASCOT), quality of aged care experience (QCE-ACC) and life satisfaction (PWI) through an online survey. Known-group validity was assessed by testing the ability to discriminate varying levels of care needs, self-reported health and quality of life.

**Results:**

Older people (aged ≥ 65 years) receiving community-aged care (*N* = 313) responded; 54.6% were female, 41.8% were living alone and 56.8% were receiving higher-level care. The QOL-ACC and its six dimensions were low to moderately and significantly correlated with the EQ-5D-5L (correlation co-efficient range, *ρ* = 0.39–0.56). The QOL-ACC demonstrated moderate and statistically significant correlations with ASCOT (*ρ* = 0.61), the QCE-ACC (*ρ* = 0.51) and the PWI (*ρ* = 0.70). Respondents with poorer self-reported health status, quality of life and/or higher-level care needs demonstrated lower QOL-ACC scores (*P* < 0.001), providing evidence of known-group validity.

**Conclusions:**

The study provides evidence of the construct validity of the QOL-ACC descriptive system. A preference-weighted value set is currently being developed for the QOL-ACC, which when finalised will be subjected to further validation assessments.

**Supplementary Information:**

The online version contains supplementary material available at 10.1007/s11136-022-03142-x.

## Introduction

In common with many other developed countries around the world, Australia’s population is ageing rapidly with sustained decline in birth rates and concomitant increased life expectancy [1]. In 2017, older Australians (aged 65 year and over) accounted for 15% (3.8 million) of the total Australian population, and by 2037, this proportion is expected to increase to 20% (6.5 million) [2]. Older people are high users of health and social care services relative to younger populations and population aging is expected to result in substantial increases in demand for aged care services and supports in future years [2, 3]. Australia has adopted long-term aged care models providing care and support outside the traditional family structure. This includes access to Federal Government subsidised aged care services either at home that help the older person with their personal care, domestic assistance and home modifications and equipment or in residential-aged care facilities (nursing homes) once they can no longer be supported adequately at home [2, 4]. There were over 1.3 million older Australians receiving some form of aged care services at June 2020, of those the majority (75%) were receiving care in their own home [5].

Although the amount of Federal Government funding for the provision of aged care services has increasing significantly in recent years [2], the aged care system in Australia and aged care systems in other countries have been criticised for the delivery of substandard care [6–8]. A recent investigation by an Australian Federal Government Royal Commission into the quality and safety of aged care found many systemic failures concluding that the current system delivers services that are too often substandard and unsafe. The Commission made a raft of policy recommendations to overhaul the system in its final report published in recent months[9]

Acknowledging that the quality and safety of aged care cannot be improved without measuring and reporting upon key quality indicators, the Royal Commission has recommended expanding the reporting for aged care providers beyond clinical indicators of care quality (e.g. pressure injury, falls, unwanted weight loss etc.) to include quality of life (QOL) to be collected and reported routinely by aged care providers from July 2023 [9]. The Quality-of-Life-Aged Care Consumers (QOL-ACC) tool has been developed for this purpose to assess older-person-specific QOL with an aged care focus and co-designed from its inception with older people using aged care services. The approach we have taken distinguishes the QOL-ACC from other instruments used in aged care settings such as the ASCOT [10] and the EQ-5D-5L [11] that were developed for broader application across social and health care rather than developed specifically for application in aged care.

The protocol for the QOL-ACC project is described in detail elsewhere [10]. The QOL-ACC instrument assesses QOL from the aged care users’ perspectives and can be integrated as a part of quality assessments by aged care providers. Once available, the preference-weighted scoring algorithm for the QOL-ACC (currently in development) will also facilitate the application of the QOL-ACC in economic evaluations to generate evidence for much needed aged care policy reforms to drive efficiency improvements and ensure resources are allocated to maximise the QOL of older people [11].

The study described in this paper is nested within a multi-phased and multi-centre study which aims to develop and validate the QOL-ACC as an instrument for quality assessment and (following the finalisation of an older-person-specific scoring algorithm, currently in development) as a preference-based instrument for economic evaluations in aged care sector [10]. The earlier phase of the project has been published elsewhere and describes in detail the identification of the QOL-ACC dimensions and its items relevant to older people receiving aged care services and the development of the final descriptive system for the QOL-ACC [12]. For a newly developed instrument such as this, it is imperative to demonstrate psychometrics robustness and evidence of validation at all stages of instrument development. Therefore, this paper aimed to describe the psychometric assessments undertaken to demonstrate the construct validity of the final QOL-ACC descriptive system in a sample of older people receiving community-aged care services.

## Methods

### Study population and data collection

Participants were older people receiving community-aged care services in their own homes via Commonwealth Home Support Programme (CHSP) or Home Care Packages (HCP) [2]. The CHSP provides entry level support services (e.g. meals, help with basic chores, home maintenance etc.) whereas HCP provides support services for more complex care (e.g. home modifications, personal care, nursing services, allied health etc.) at four levels (1 = basic to 4 = high) [13]. All study participants were recruited through an online panel as face-to-face interviewer-facilitated data collection in individual’s own homes was not possible due to COVID-19 restrictions at the time of the study. The online panel utilised for this study is an Australia-wide online panel network comprising of over 10,000 nationally representative Australians aged 65 years and older. Panel members who were aged 65 years and over, able to read and respond in English, living in Australia and currently receiving CHSP or HCP services were invited to take part in the survey. Quotas for recruitment were set by age, gender and types of care to ensure broad representation. All study participants provided online consent prior to completing the survey. The study was approved by the Human Research Ethics Committee at Flinders University (application numbers 8399 and 2201) and adheres to the tenets of the Declaration of Helsinki.

### The QOL-ACC

The content of the QOL-ACC was identified from in-depth qualitative interviews with older people receiving aged care services exploring what quality of life meant to them supplemented by a comprehensive literature review [12, 14]. A set of draft items were developed across QOL-ACC dimensions (mobility, pain management, independence, emotional well-being, social connections and activities). A mixed method approach using a traffic light system was used to integrate qualitative (face validity) and quantitative (psychometric assessments) evidence to develop the final descriptive system for the QOL-ACC [15]. The final QOL-ACC descriptive system has 6 dimensions with single item across all the dimensions rated on a 5 a five-point frequency scale (Table 1).

**Table 1:** The final Quality-of-Life-Aged Care Consumer (QOL-ACC) descriptive system

Dimension	Item*
Mobility	I am able to get around as much as I want to *(with the use of mobility aids e.g. wheelchair, walker, stick if you use them)*
Pain management	When I experience pain, it is well managed
Emotional well-being	I am generally happy
Independence	I have as much independence as I want
Social connection	I have good social relationships with family and friends
Activities	I have leisure activities/hobbies I enjoy

*Levels identical to all dimensions: All of the time, Most of the time, Some of the time, A little of the time, None of the time

### Other instruments and data

Survey respondents also completed four other instruments, two global items of quality of life and health status rated on a 5-point response scale (Poor to Excellent) and socio-demographic details. Using postcode data (geographical areas of their residents), two indices (Index of Relative Socio-economic Advantage and Disadvantage, IRSAD and Index of Education and Occupation, IEO) of socio-economic well-being were estimated using methodology described by the Australian Bureau of Statistics [16].

***The EQ-5D-5L*** is a generic preference-based HRQOL utility instrument which provides descriptions and valuations of five dimensions (mobility, self-care, usual activities, pain/discomfort and anxiety/depression) of health status on a 5-level scale (no problems to extreme problems). It is one of the most widely used utility instruments and various sets of preference-based utility values have been developed across cross-national general population samples [17]. For this study, we used the Australian preference weights developed by Norman et al. [18]. The EQ-VAS is a vertical visual analogue scale of self-reported health which ranges from 0 (worst possible health one can imagine) to 100 (best possible health one can imagine). The EQ-VAS scores were used as a standalone measure of health reflecting the respondents’ own judgement of their health status.

***The ASCOT**** (Adult Social Care Outcome Tool)* is a preference based social-care-related QOL instrument [19, 20]. It has eight domains: personal cleanliness and comfort, food and drink, control over daily life, safety, accommodation cleanliness and comfort, social participation and involvement, occupation and dignity. Each domain is framed as “which of the following statements best describes...” and rated on a four response levels representing four different outcome status (Ideal, the preferred situation; No needs, where needs are made; Some needs, where there are needs but no immediate/long-term health implications; High needs, where needs have immediate and long-term health implications),[20] and the ASCOT total preference-weighted scores for English general population range from -0.17 to 1.0 with higher scores representing better QOL [19].

***The QCE-ACC**** (Quality of Care-Aged Care Consumers)* is a preference-based weighted measure of aged care-specific quality of care. The QCE-ACC was developed from a study commissioned by the Royal Commission into Aged Care Quality and Safety in Australia [21]. The QCE-ACC has 6 dimensions (respect and dignity, services and supports, decision making, staff skills and training, social relationships, complaints) rated across 5-response options (“all of the time” to “none of the time”). It has been validated in a separate sample of aged care recipients in the community and residential care settings [22].

***The PWI**** (Personal Well-being Index)* is a measure of subjective well-being [23]. It has seven dimensions of life satisfaction: standard of living, health, achievement in life, relationships, safety, community connectedness and future security. These seven domains are the core set of items forming a composite score and each dimension can also be scored separately. Respondents were asked to rate their level of satisfaction in each domain on an 11-point end-defined scale anchored by “not at all satisfied at all” to “completely satisfied”. There are two additional PWI items (Global life satisfaction and Spirituality or Religion) which are not included in the PWI composite scores, but can be scored separately and these items were also used to assess the construct validity of the QOL-ACC.

### Construct validity

Validity assessments for the QOL-ACC were guided by the Consensus-based Standards for the Selection of Health Measurement Instruments (COSMIN) checklists [24, 25]. Two important validity assessments are content and construct validity of a descriptive system. Content validity is the extent to which the content of the instrument appears to include full scope of the constructs relevant to the target population. Our group has published two papers describing a multi-stage rigorous process of qualitative interviews to identify content of the QOL-ACC and face validity assessments of draft items with older people using aged care services to inform content validity of the QOL-ACC [12, 15].

Construct validity is the extent to which an instrument validly measures the construct it purports to measure. We assessed a range of indicators of construct validity in relation to convergent and known-group validity. Convergent validity is a type of construct validity which examines whether an instrument correlates with other instruments to the degree that is expected. Assessing convergent validity is an iterative process: the more hypotheses tested, the stronger the evidence towards the instrument being valid. In the absence of a gold standard, the convergent validity of the QOL-ACC and its dimensions was assessed against two existing validated preference-based instruments (EQ-5D-5L, ASCOT) [11, 26], a measure of quality of aged care (QCE-ACC) and a measure of life satisfaction (PWI). A significant but low to medium (correlation range of > 0.30 to 0.70) correlation is indicative of good convergent validity, with related constructs expected to have a stronger correlation than unrelated constructs [27]. A series of 21 key hypotheses were developed to appraise convergent validity of the QOL-ACC against other constructs (Table 2). For example, we hypothesised that the QOL-ACC would be more closely associated with PWI (a psychosocial component of QOL) and ASCOT (social-care-related QOL) than EQ-5D-5L (health-related QOL). Convergent validity was perceived as adequate if more than 75% of the hypothesised relationships, in terms of the directions and strengths of correlations, were supported by the analysis results [27, 28]. Known-group validity is the extent to which an instrument discriminates between groups known to be different. Known-group validity was assessed by testing the QOL-ACC’s ability to discriminate the varying levels of aged care needs, aged care quality, self-reported health and quality of life.

**Table 2:** A priori hypothesised association between the Quality-of-Life-Aged Care Consumer (QOL-ACC) instrument, its dimensions and other related constructs

Hypothesis No.	Instrument/Dimension	Expected relationships with the QOL-ACC and its dimensions*	Achieved	
QOL-ACC overall and other instruments				
1	EQ-5D-5L	Generic health-related quality of life as measured by the EQ-5D-5L and older-person-specific quality of life as measured by the QOL-ACC are related constructs. Therefore, it was expected that the EQ-5D-5L and QOL-ACC would demonstrate a positive, moderate and significant correlation	Yes	
2	ASCOT overall	Social-care-related quality of life as measured by the ASCOT and older-person-specific quality of life in aged care settings as measured by the QOL-ACC are related constructs. Therefore, it was expected that both the ASCOT and QOL-ACC overall scores would demonstrate a positive, moderate and significant correlation. The correlation would be stronger than with generic health-related quality of life (EQ-5D-5L)	Yes	
3	PWI overall	Older people reporting higher satisfaction for subjective well-being would also be expected to report better quality of life. Therefore, it was expected that personal well-being (a mental component of quality of life) as measured by the PWI (a multi-domain life satisfaction scale) would demonstrate a positive, moderate and significant correlation with the older-person-specific QOL-ACC measure. The correlation between the QOL-ACC and PWI was expected to be stronger than between the QOL-ACC and ASCOT or EQ-5D-5L	Yes	
4	PWI global item life satisfaction	Individuals with a higher life satisfaction may have a better perceived quality of life and self-reported health. Therefore, it was expected that life satisfaction as measured by a global item of life satisfaction would demonstrate a positive and significant correlation with the QOL-ACC. The correlation between the PWI global item of life satisfaction and the QOL-ACC was expected to be stronger than between the QOL-ACC and EQ-VAS (measure of health)	Yes	
5	EQ-VAS	Individuals who perceive to have better health are expected to have a better perceived quality of life. Therefore, it was expected the EQ-VAS would demonstrate a high and significant correlation with the QOL-ACC	No	
6	QCE-ACC	Individuals who self-report a better quality of aged care experience are more likely to have their care needs addressed appropriately leading to a higher perceived quality of life. Therefore, it was expected that the quality of aged care experience as measured by the QCE-ACC would demonstrate a positive and significant correlation with quality of life as measured by the QOL-ACC. However, as these two constructs (quality of care and quality of life) are reflecting different concepts, it was expected that the strength of the correlation between the QOL-ACC and QCE-ACC would be positive but weaker than the correlations between QOL-ACC and PWI, EQ-5D-5L and ASCOT	Yes	
QOL-ACC dimensions				
7	ASCOT domain of control over daily living	*QOL-ACC dimensions of Independence:* The ASCOT domain of control is reflective of individual’s control over activities they want do and enjoy. Therefore, it was expected that the ASCOT domain of control would demonstrate a positive and moderate correlation with the QOL-ACC dimension of Independence		Yes
8	ASCOT domain of occupation	*QOL-ACC dimension of Independence:* The ASCOT occupation domain captures whether individuals have access to undertake activities that are valued, enjoyable and meaningful to them. Therefore, it was expected that this domain would demonstrate a positive and moderate correlation with the QOL-ACC dimension of Independence		Yes
9	EQ-5D-5L	*QOL-ACC dimension of Mobility:* The EQ-5D-5L has a mobility dimension. Therefore, it was expected that the QOL-ACC dimension of Mobility would demonstrate a positive and moderate correlation with the EQ-5D-5L		Yes
10	ASCOT domain of control over daily living	*QOL-ACC dimension of Mobility:* The ASCOT domain of control is reflective of individual’s control over activities they want to do and enjoy. Therefore, it was expected that the ASCOT domain of control would demonstrate a moderate and significant correlation with the QOL-ACC dimension of Mobility. The strength of correlation was expected to be weaker than the correlation between the ASCOT domain of control and the QOL-ACC dimension of Mobility		Yes
11	ASCOT domain of social participation and involvement	*QOL-ACC dimension of Social connections:* It was expected that the ASCOT domain of social participation would demonstrate positive and moderate correlation with the QOL-ACC dimension of Social connection		Yes
12	PWI domain of Personal Relationships	*QOL-ACC dimension of Social connections:* The PWI domains of Personal relationship and the QOL-ACC dimension of Social Connectedness are capturing a similar construct of social connection. Therefore, the PWI domain of Personal Relationship and the QOL-ACC dimension of Social Connection were expected to have a positive and moderate correlation		Yes
13	PWI domain of Community Connectedness	*QOL-ACC dimension of Social connections:* The PWI domain of community captures the construct of being engaged with the community. Hence, it was expected this domain would have a positive and moderate correlation with the QOL-ACC domain of Social connections		Yes
14	PWI domain of personal safety	*QOL-ACC dimension of Social connections:* Individuals who feel safe have more social engagement, a stable relationship and have little or no issues engaging in leisure activities and hobbies. Therefore, it was expected that this domain would show a positive and moderate correlation with the QOL-ACC dimension of Social Connection		Yes
15	PWI domain of achievement	*QOL-ACC dimension of Emotional well-being:* Individuals who are satisfied with their current life achievements may have better perceived mental health status and quality of life. Therefore, a positive and moderate correlation was expected with the QOL-ACC dimension of Emotional well-being with the PWI domain of achievement		Yes
16	PWI domain of standard of living	*QOL-ACC dimension of Emotional well-being:* Individuals who are more satisfied with their standard of living may have a better perceived emotional well-being and quality of life. Therefore, it was expected that the PWI domain of standard of living would have a positive and moderate correlation with the QOL-ACC dimension of Emotional well-being		Yes
17	PWI domain of future Security	*QOL-ACC dimension of Emotional well-being*: Individuals who are satisfied with their future security may have a better emotional status and more positive social connections than those who are less satisfied. Therefore, it was expected that the PWI domain of future security would have a positive, moderate and significant association with the QOL-ACC dimension of Emotional well-being		Yes
18	PWI domain of personal health	*QOL-ACC dimension of Pain management:* Individuals who are more satisfied with their personal health have a better perceived quality of life and better managed pain. Therefore, it was expected that the PWI domain of personal Health would have a positive, high and significant correlation with the QOL-ACC dimension of Pain management		No
19	EQ-5D VAS	*QOL-ACC dimension of Pain management:* Individuals who perceive to have a better health are expected to have better pain management. Therefore, it was expected the EQ-VAS would demonstrate a positive, high and significant correlation with the QOL-ACC dimension of Pain management		No
20	PWI domain of achievement	*QOL-ACC dimension of Activity:* Individuals who are satisfied with their current life achievements may have better perceived mental health status and quality of life. Therefore, a positive and moderate correlation was expected with the QOL-ACC dimension of Activity with the PWI domain of achievement		Yes
21	ASCOT domain of occupation	*QOL-ACC dimension of Activity:* The ASCOT occupation domain captures whether individuals have access to undertake activities that are valued, enjoyable and meaningful to them. Therefore, it was expected that the ASCOT domain of occupation would demonstrate a positive and moderate correlation with the QOL-ACC dimension of Activity		Yes

*EQ-5D-5L* Euroqol 5 dimensions 5-level instrument, *ASCOT* Adult Social Care Outcomes Toolkit, *QCE-ACC* Quality of Care Experience-Aged Care Consumer, *PWI* Personal Well-being Index-Adult

^*^Desired correlations between the QOL-ACC/its dimensions with other related constructs were low to moderate

### Statistical analysis

Descriptive data were analysed using STATA Version 15.1, Stata Corp LLC, Texas, USA. The Winsteps software (Ver 5.0.0) was used to conduct Rasch analysis on the final descriptive QOL-ACC data and to produce interval level overall and dimensional level scorings [29]. Rasch analysis is an item response theory-based scaling psychometric technique that unlike traditional psychometric methods (e.g. Classical Test Theory) can provide insights into important psychometric properties both at the scale and item level such as adequacy of category functioning, monotonicity of category option use, item fit statistics, dimensionality, item bias etc. We used quantitative evidence from Rasch analysis to identify items possessing the best psychometric properties to develop the final descriptive system of the QOL-ACC from an initial item pool set [15]. Further, as the QOL-ACC descriptive system is a new instrument under construction, Rasch-based psychometric properties guided item selection provided important evidence to take it to the next stages of its development and validation. Further, Rasch analysis converts each categorical dimension onto a unique unidimensional latent scale to estimate interval level scoring for both the items and respondents on the same continuum scale. Rasch analysis has been widely applied in the development and validation of instruments to measure health-specific quality-of-life constructs [30–33]. Given that the QOL-ACC has 6 dimensions, we used the Rasch Partial Credit Model with conditional maximum likelihood to estimate respondents’ parameters. Similarly, a Rasch model based methodological approach was applied by the EuroQoL group for the EQ-5D-5L initial validations [33].

The QOL-ACC Rasch scores in logits were rescaled from 0 (worse score) to 100 (best score) to facilitate ease of interpretation. We used Pearson’s Chi-squared tests for categorical variables and Kruskal Wallis Test to assess the difference in the EQ-5D-5L, EQ-VAS, ASCOT, PWI and QCE-ACC scores by the QOL-ACC dimension levels (test of monotonicity: to indicate that the scores on other instruments increased by response levels across the QOL-ACC dimensions). Person’s Chi-squared test was used to assess whether the distribution of the QOL-ACC response categories across its six dimensions was different between respondents using CHSP and HCP. For convergent validity (to assess the extent to which the QOL-ACC and other instruments measure related constructs), Spearman’s rank absolute correlation co-efficient (*P* values) were produced based on the instruments scores distribution assessments (Appendix S1). The size of correlation coefficients is interpreted as negligible (0.00 to 0.30), low (> 0.30 to 0.50), moderate (> 0.50 to 0.70) and high (> 0.70 to 0.90). These analyses were complemented by locally weighted scatterplot smoothing (LOWESS) techniques. The LOWESS is a form of non-parametric regression which plots a line of central tendency between two measures on a scatterplot (visually) to demonstrate relationship across all the possible score ranges without making assumption about the actual relationships [34]. For known-group validity, Kruskal–Wallis test was used to test differences between the multiple groups. Dunn’s test was carried out following Kruskal–Wallis test for multiple pairwise comparison between the groups [35]. A total of 21 hypotheses were tested to assess the construct validity of the QOL-ACC. To adjust for multiple testing, we used the Bonferroni technique to set the significance threshold at *P* ≤ 0.05/21 = 0.002.

## Results

### Sample description

In total, 1878 older people (≥ aged 65 years) living in the community were initially approached; however, 1479 did not meet the inclusion criteria (i.e. not currently receiving aged care services) and were screened out leaving 399 individuals who attempted the survey. Out of 399, 313 (78.4%) older people fully completed the survey. Of those who completed 54.6% were female and 76.4% were Australian born. Participants ranged in age from 65 to 91 years (mean 74.5 years, ± 5.9) and slightly more than half of the study sample (50.5%) were between 65 and 74 years old (Table 3). Approximately half of the study respondents (50.5%) lived with their spouse or partner and 41.9% lived alone at the time of the survey. Only 14.7% of survey respondents described their overall health as either “excellent” or “very good” whilst 32.9% described their overall QOL as either “excellent” or “very good” (Table 3). More participants were receiving a lower level of care package such as the CHSP (38.3%), or HCP level 1 (18.5%) and level 2 (21.7%) than the higher-level HCPs (level 3 and level 4). Almost half of the respondents were in the lower quintiles of SEIFA index for IRSEAS (46.4%) and more than one third were in the lower quintiles of SEIFA index for IEOA (35.1%) (Table 3). In comparison with national data describing the socio-demographic characteristics of older Australians using CHSP and HCP nationally in 2021, our study cohort also had more representation from females (study cohort vs national cohort; 54.6% vs 65.4%), aged < 85 years older (study cohort vs national cohort; 93.6% vs 63.8%) and using lower-level support services i.e. CHSP to HCP2 (study cohort vs national cohort; 78.5% vs 92.2%) (Table 3) [5].

**Table 3:** Socio-demographic characteristics of the respondents

Variables	*N* = 313 (100%)	People using CHSP and HCP nationally at 30^th^ June 2021
Gender, *N* (%)		
Male	142 (45.4)	34.6%
Female	171 (54.6)	65.4%
Age, *N* (%)		
65–74	158 (50.5)	21.5%
75–84	135 (43.1)	42.3%
85+	20 (6.4)	36.2%
Mean Age (SD)	75 (5.9)	
Median Age (IQR)	74 (70–78)	
Range	65–91	
*Care Packages and Levels, N (%)*		
Commonwealth Home Support Programme (CHSP)	120 (38.3)	84.0%
Home Care Package—Level 1	58 (18.5)	1.9%
Home Care Package—Level 2	68 (21.7)	6.3%
Home Care Package—Level 3	25 (8.0)	3.7%
Home Care Package—Level 4	27 (8.6)	4.0%
Unsure	15 (4.8)	
Living arrangements, *N* (%)		
Living alone	131 (41.9)	
Living with spouse/partner	158 (50.5)	
Living with relatives	16 (5.1)	
Living with others (not relatives)	8 (2.6)	
Informal carer availability		
Yes	125 (39.9)	
No	188 (60.1)	
Country of birth, *N* (%)		
Australia	238 (76.4)	
UK	33 (10.5)	
Others	42 (13.4)	
Highest educational qualification, *N* (%)		
No qualifications	42 (13.4)	
Completed high school	95 (30.4)	
Undergraduate degree/Professional qualification	109 (34.8)	
Postgraduate qualification	44 (14.1)	
Other	23 (.3)	
Hours of support services per week, *N*(%)		
≤ 2 h	211 (67.4)	
3–4 h	44 (14.1)	
5-9 h	34 (10.9)	
≥ 10 h	24 (7.7)	
Co-contribution for the care they receive, *N*(%)		
None	87 (27.8)	
Make a small contribution	182 (58.2)	
Make a large contribution	18 (5.7)	
Pay for all of my care	26 (8.3)	
Types of services being received**, *N*(%)		
Meals or help with cooking	49 (15.6)	
Cleaning	277 (88.5)	
Shopping	55 (17.6)	
Transportation	76 (24.3)	
Gardening	123 (39.3)	
Personal care	30 (9.6)	
Home nursing	15 (4.8)	
Group social activities	17 (5.4)	
Respite care in the home	10 (3.2%)	
Others	29 (9.3%)	
Self-reported health, *N* (%)		
Excellent	1 (0.3)	
Very good	45 (14.4)	
Good	104 (33.2)	
Fair	121 (38.7)	
Poor	42 (13.4)	
Self-reported quality of life, *N*(%)		
Excellent	14 (4.5)	
Very good	89 (28.4)	
Good	132 (42.2)	
Fair	70 (22.4)	
Poor	8 (2.6)	
SEIFA-IRSEAS quintiles, *N*(%)		
1 (least advantaged)	59 (24.9)	
2	51 (21.5)	
3	62 (26.2)	
4	49 (20.7)	
5 (most advantaged)	16 (6.7)	
SEIFA- IEO quintiles, *N*(%)		
1 (least advantaged)	59 (18.8)	
2	51 (16.3)	
3	62 (19.8)	
4	49 (15.6)	
5 (most disadvantaged)	92 (29.4)	
EQ-5D-5L (mean ± SD)	0.53 ± 0.31	
ASCOT (mean ± SD)	0.68 ± 0.12	
QCE-ACC (mean ± SD)	0.89 ± 0.13	
PWI (mean ± SD)	69.4 ± 18.8	

Social Economic Indices for Areas- Index of Relative Socio-Economic Advantage and Disadvantage; SEIFA-IEO: Social Economic Indices for Areas- Index for Education and Occupation

*ASCOT* The Adult Social Care Outcome Toolkit, *QCE-ACC* Quality of Care-Aged Care Consumers, *PWI* Personal Well-being Index

**Individual might be receiving more than one service types and the percentage for a specific service type was estimated out of *N* = 313

The responses to the final 6-dimension QOL-ACC are summarised in Table 4. With the notable exception of the pain management dimension, the majority of respondents (> 70%) indicated good QOL across QOL-ACC dimensions. Three dimensions (mobility, emotional well-being and independence) had a significant difference in the distribution of responses between those using CHSP and HCP reflective of the increasing dependency levels associated with HCPs relative to CHSP which provides an entry level of care and support. This was not observed in the remaining three dimensions (pain management, social connections and activities) between CHSP and HCP groups.

**Table 4:** Responses to the Quality-of-Life-Aged Care Consumer (QoL-ACC) by all the respondents and the respondents stratified by types of care packages

	Total, *N* = 313 (100%)	CHSP, *N* = 120 (100%)	HCP, *N* = 178 (100%)	P*
*I am able to get around as much as I want to*				
All of the time	134 (42.8)	71 (59.2)	58 (32.6)	< 0.001 (25.2)
Most of the time	108 (34.5)	35 (29.2)	67 (37.6)	
Some of the time	34 (10.9)	9 (7.5)	22 (12.4)	
A little of the time	29 (9.3)	4 (3.3)	24 (13.5)	
None of the time	8 (2.6)	1 (0.8)	7 (3.9)	
*When I experience pain, it is well managed*	*N* = 163#	*N* = 72	*N* = 86	0.52 (3.23)
All of the time	19 (11.7)	10 (13.9)	9 (10.5)	
Most of the time	63 (38.6)	32 (44.4)	29 (33.7)	
Some of the time	59 (36.2)	21 (29.2)	35 (40.7)	
A little of the time	15 (9.2)	6 (8.3)	9 (10.5)	
None of the time	7 (4.3)	3 (4.2)	4 (4.6)	
*I am generally happy*				
All of the time	67 (21.4)	20 (16.7)	44 (24.7)	0.05 (9.52)
Most of the time	166 (53.0)	74 (61.7)	83 (46.6)	
Some of the time	58 (18.5)	22 (18.3)	34 (19.1)	
A little of the time	20 (6.4)	4 (3.3)	15 (8.4)	
None of the time	2 (0.6)	0 (0)	2 (1.1)	
*I have as much independence as I want*				
All of the time	130 (41.5)	60 (50.0)	65 (36.5)	0.02 (12.1)
Most of the time	110 (35.1)	43 (35.8)	60 (33.7)	
Some of the time	56 (17.9)	15 (12.5)	39 (21.9)	
A little of the time	14 (4.5)	2 (1.7)	11 (6.2)	
None of the time	3 (1.0)	0 (0)	3 (1.7)	
*I have good social relationships with family and friends*				
All of the time	152 (48.6)	67 (55.8)	79 (44.4)	0.39 (4.14)
Most of the time	99 (31.6)	32 (26.7)	60 (33.7)	
Some of the time	31 (9.9)	11 (9.2)	19 (10.7)	
A little of the time	23 (7.4)	8 (6.7)	14 (7.9)	
None of the time	8 (2.6)	2 (1.7)	6 (3.4)	
*I have leisure activities/hobbies I enjoy*				
All of the time	93 (29.7)	39 (32.5)	49 (27.5)	0.27 (5.18)
Most of the time	90 (28.7)	40 (33.3)	46 (25.8)	
Some of the time	65 (20.8)	22 (18.3)	40 (22.5)	
A little of the time	45 (14.4)	14 (11.7)	29 (16.3)	
None of the time	20 (6.4)	5 (4.2)	14 (7.9)	

*CHSP* Commonwealth Home Support Programme; *HCP* Home Care Packages

^*^Pearson’s chi-squared was used to generate *P* values

^#^The responses were lower for the Pain management dimension because the psychometric assessment survey was conducted in two stages. In the first stage, 5 dimensions other than pain was completed by 313 older people (≥ 65 years) (Table 3) receiving community-aged care services. In the second stage of data collection, a subset (*N* = 165) of stage 1 psychometrics survey respondents completed the draft pain items

### Construct validity

#### Convergent validity of the QOL-ACC

LOWLESS graphs (see Appendix S2) and Table 5 suggested that the QOL-ACC scores had higher convergent validity (also see Table 2 for priori hypotheses) with the EQ-5D-5L (*ρ* = 0.56, *P* < 0.001; hypothesis 1, ASCOT (*ρ* = 0.61, *P* < 0.001, hypothesis 2 and PWI (*ρ* = 0.70, *P* < 0.001, hypothesis 3),. The QOL-ACC also demonstrated high correlations with the PWI global item of life satisfaction and the strength of the correlation was higher than the correlation between the QOL-ACC and the EQ-5D VA (hypothesis 4). The QOL-ACC did not show a high correlation with the EQ-VAS as expected (hypothesis 5 was not met).

**Table 5:** Mean scores (standard deviation) in other instruments by the QOL-ACC dimensions

QOL-ACC Dimension and levels (*N*)	EQ-5D-5L	EQ-5D-VAS	QCE-ACC	ASCOT	PWI
*I am able to get around as much as I want to*					
All of the time (134)	0.71 (0.18)	71.6 (18.0)	0.90 (0.13)	0.72 (0.09)	75.1 (17.7)
Most of the time (108)	0.45 (0.30)	59.4 (20.8)	0.89 (0.12)	0.69 (0.10)	69.1 (16.5)
Some of the time (34)	0.38 (0.28)	56.5 (20.1)	0.85 (0.13)	0.64 (0.11)	62.8 (18.0)
A little of the time/ None of the time (38)	0.25 (0.37)	49.2 (23.5)	0.82 (0.16)	0.54 (0.18)	55.5 (20.5)
P*	< 0.001	< 0.001	0.0006	< 0.001	< 0.001
*When I experience pain, it is well managed^*					
All of the time (19)	0.70 (0.30)	67.9 (22.7)	0.90 (0.10)	0.68 (0.17)	71 .7 (17.8)
Most of the time (63)	0.64 (0.26)	69.4 (18.1)	0.91 (0.11)	0.69 (0.12)	73.6 (18.6)
Some of the time (59)	0.50 (0.25)	60.4 (20.8)	0.89 (0.11)	0.68 (0.10)	68.3 (17.3)
A little of the time/none of the time (22)	0.19 (0.40)	44.9 (17.4)	0.83 (0.16)	0.62 (0.17)	59.5 (19.2)
P*	< 0.001	< 0.001	***0.282#***	0.011	***0.28#***
*I am generally happy*					
All of the time (67)	0.68 (0.25)	71.7 (19.2)	0.94 (0.10)	0.74 (0.07)	81.6 (16.3)
Most of the time(166)	0.56 (0.26)	66.4 (19.7)	0.89 (0.11)	0.70 (0.10)	72.7 (15.1)
Some of the time (58)	0.34 (0.33)	49.8 (18.7)	0.82 (0.16)	0.61 (0.09)	55.7 (15.1)
A little of the time/ none of the time (22)	0.26 (0.35)	47.0 (23.8)	0.81 (0.18)	0.53 (0.15)	43.3 (14.0)
P*	< 0.001	< 0.001	< 0.001	< 0.001	< 0.001
*I have as much independence as I want*					
All of the time (130)	0.64 (0.28)	69.7 (20.1)	0.92 (0.10)	0.74 (0.81)	76.3 (17.3)
Most of the time (110)	0.53 (0.28)	64.9 (19.9)	0.86 (0.15)	0.68 (0.10)	72.0 (15.1)
Some of the time (56)	0.37 (0.28)	50.9 (19.2)	0.86 (0.10)	0.59 (0.15)	55.8 (16.8)
A little of the time/ None of the time (17)	0.15 (0.35)	41.8 (18.1)	0.75 (0.21)	0.50 (0.14)	43.0 (12.3)
P*	< 0.001	< 0.001	< 0.001	< 0.001	< 0.001
*I have good social relationships with family and friends*					
All of the time (152)	0.62 (0.25)	68.4 (19.3)	0.93 (0.09)	0.73 (0.07)	79.1 (14.1)
Most of the time (99)	0.48 (0.32)	61.2 (21.9)	0.85 (0.14)	0.66 (0.11)	64.8 (16.7)
Some of the time (31)	0.44 (0.31)	56.8 (21.0)	0.80 (0.17)	0.58 (0.18)	56.5 (17.9)
A little of the time (31)	0.33 (0.39)	49.6 (22.3)	0.80 (0.15)	0.58 (0.13)	48.8 (16.9)
P*	< 0.001	< 0.001	< 0.001	< 0.001	< 0.001
*I have leisure activities/hobbies I enjoy*					
All of the time (93)	0.60 (0.30)	69.9 (20.2)	0.95 (0.08)	0.73 (0.84)	80.0 (13.6)
Most of the time (90)	0.60 (0.26)	65.6 (21.4)	0.89 (0.13)	0.71 (0.08)	74.1 (15.7)
Some of the time (65)	0.51 (0.28)	61.3 (19.2)	0.88 (0.12)	0.66 (0.13)	64.1 (18.9)
A little of the time/None of the time (65)	0.34 (0.34)	51.8 (20.8)	0.78 (0.16)	0.59 (0.15)	52.9 (15.9)
P*	< 0.001	< 0.001	< 0.001	< 0.001	< 0.001

@ Lowest two levels (A little of the time and None of the time) were collapsed for analysis due to low cell counts. *Kruskal Wallis Test; # statistically not significant

^ The psychometrics survey was conducted in two stages. In the first stage, 5 dimensions (Mobility, Emotional well-being, Independence, Social connection, Activities) were completed by *N* = 313. In the second stage, a subset (*N* = 165) of the stage 1 psychometrics survey respondents completed the Pain management dimension

**Table 6:** Relationship between the Quality-of-Life-Aged Care Consumer-(QOL-ACC) and other instruments *(Construct validity)*

	QOL-ACC, Spearman’s rho correlation co-efficient (*P* values)						
	Overall	Mobility	Pain	Independence	Emotional well-being	Social connections	Activity
EQ-5D-5L	0.56 (< 0.001)	0.53 (< 0.001)	0.42 (< 0.001)	0.44 (< 0.001)	0.42 (< 0.001)	0.31 (< 0.001)	0.29 (< 0.001)
EQ-5D VAS	0.48 (< 0.001)	0.37 (< 0.001)	0.35 (< 0.001)	0.38 (< 0.001)	0.38 (< 0.001)	0.26 (< 0.001)	0.31 (< 0.001)
*Adult Social Care Outcomes Toolkit (ASCOT)*							
Overall	0.61 (< 0.001)	0.36 (< 0.001)	**0.15 (0.06)**	0.51 (< 0.001)	0.46 (< 0.001)	0.46 (< 0.001)	0.42 (< 0.001)
Control over daily life	0.52 (< 0.001)	0.41 (< 0.001)	**0.15 (0.06**)	0.57 (< 0.001)	0.34 (< 0.001)	0.28 (< 0.001)	0.30 (< 0.001)
Personal cleanliness and comfort	0.42 (< 0.001)	0.33 (< 0.001)	**0.07 (0.35)**	0.39 (< 0.001)	0.27 (< 0.001)	0.32 (< 0.001)	0.29 (< 0.001)
Food & drink	0.38 (< 0.001)	0.31 (< 0.001)	**0.03 (0.69)**	0.26 (< 0.001)	0.27 (< 0.001)	0.27 (< 0.001)	0.26 (< 0.001)
Safety	0.38 (< 0.001)	0.25 (< 0.001)	0.23 (0.003)	0.28 (< 0.001)	0.31 (< 0.001)	0.28 (< 0.001)	0.24 (< 0.001)
Social participation and involvement	0.57 (< 0.001)	0.28 (< 0.001)	**0.08 (0.30)**	0.43 (< 0.001)	0.38 (< 0.001)	0.50 (< 0.001)	0.44 (< 0.001)
Occupation	0.59 (< 0.001)	0.34 (< 0.001)	0.23 (0.003)	0.52 (< 0.001)	0.39 (< 0.001)	0.38 (< 0.001)	0.45 (< 0.001)
Accommodation cleanliness and comfort	0.42 (< 0.001)	0.31 (< 0.001)	**0.09 (0.23)**	0.38 (< 0.001)	0.27 (< 0.001)	0.32 (< 0.001)	0.25 (< 0.001)
Dignity	0.18 (0.001)	**0.06 (0.32)**	**0.01 (0.88)**	0.18 (0.002)	0.20 (0.0003)	0.15 (0.006)	0.15 (0.007)
QCE-ACC	0.51 (< 0.001)	0.22 (< 0.001)	**0.13 (0.09)**	0.36 (< 0.001)	0.35 (< 0.001)	0.43 (< 0.001)	0.47 (< 0.001)
*Personal Well-being Index (PWI)*							
Overall	0.70 (< 0.001)	0.34 (< 0.001)	0.23 (0.003)	0.46 (< 0.001)	0.58 (< 0.001)	0.57 (< 0.001)	0.54 (< 0.001)
Living standard	0.56 (< 0.001)	0.23 (< 0.001)	**0.14 (0.08)**	0.39 (< 0.001)	0.49 (< 0.001)	0.45 (< 0.001)	0.45 (< 0.001)
Health	0.54 (< 0.001)	0.37 (< 0.001)	0.35 (< 0.001)	0.41 (< 0.001)	0.41 (< 0.001)	0.33 (< 0.001)	0.36 (< 0.001)
Achievement	0.67 (< 0.001)	0.34 (< 0.001)	0.28 (0.0004)	0.44 (< 0.001)	0.53 (< 0.001)	0.48 (< 0.001)	0.53 (< 0.001)
Relationships	0.55 (< 0.001)	0.14 (0.01)	**0.11 (0.16)**	0.25 (< 0.001)	0.50 (< 0.001)	0.55 (< 0.001)	0.46 (< 0.001)
Feel safe	0.50 (< 0.001)	0.29 (< 0.001)	**0.10 (0.21)**	0.36 (< 0.001)	0.39 (< 0.001)	0.39 (< 0.001)	0.36 (< 0.001)
Community	0.58 (< 0.001)	0.25 (< 0.001)	**0.09 (0.27)**	0.40 (< 0.001)	0.45 (< 0.001)	0.50 (< 0.001)	0.47 (< 0.001)
Future security	0.57 (< 0.001)	0.26 (< 0.001)	0.17 (0.03)	0.38 (< 0.001)	0.48 (*P* < 0.001)	0.47 (< 0.001)	0.44 (< 0.001)
PWI_global	0.66 (< 0.001)	0.32 (< 0.001)	0.28 (0.0003)	0.47 (< 0.001)	0.61 (< 0.001)	0.48 (< 0.001)	0.46 (< 0.001)
PWI_religion	0.43 (< 0.001)	0.23 (< 0.001)	0.13 (0.10)	0.29 (< 0.001)	0.39 (< 0.001)	0.38 (< 0.001)	0.30 (< 0.001)

The QOL-ACC also demonstrated high correlations with all seven domains of the PWI (*ρ* ranges from 0.50 to 0.67, all *P* < 0.001). The QOL-ACC scores also demonstrated adequate convergent validity with the QCE-ACC (*ρ* = 0.51, *P* < 0.001; hypothesis 6) but as expected this association was not as high as found with other instruments as the QCE-ACC focuses on the quality of care experience rather than quality-of-life/well-being outcomes (Table 6). It also exhibited relatively high correlations with the ASCOT domains of occupation (*ρ* = 0.59, *P* < 0.001), social (*ρ* = 0.57, *P* < 0.001) and control (*ρ* = 0.51, *P* < 0.001). The ASCOT domain of dignity (*ρ* = 0.18, *P* < 0.001) demonstrated low correlation with the QOL-ACC.

#### Convergent validity of the QOL-ACC dimensions

### Independence

The independence dimension had a stronger correlation with the ASCOT (*ρ* = 0.51, *P* < 0.001) and its domains of control (*ρ* = 0.57, *P* < 0.001, hypothesis 7) and occupation (*ρ* = 0.52, *P* < 0.001, hypothesis 8). It had similar correlations with the PWI global item of life satisfaction (*ρ* = 0.47, *P* < 0.001), PWI domains of achievement (*ρ* = 0.44, *P* < 0.001) and health (*ρ* = 0.41, *P* < 0.001) (Table 6).

### Mobility

The mobility dimension had the highest correlation with the EQ-5D-5L (*ρ* = 0.53, *P* < 0.001, hypothesis 9) and the ASCOT domain of control (*ρ* = 0.41, *P* < 0.001, hypothesis 10).

### Social connections

The social connections dimension demonstrated a strong correlation with the ASCOT domain of social (*ρ* = 0.50, *P* < 0.001, hypothesis 11), PWI domains of relationships (*ρ* = 0.55, *P* < 0.001, hypothesis 12) and PWI domain of community (*ρ* = 0.50, *P* < 0.001, hypothesis 13) (Table 6).

### Emotional well-being

The emotional well-being dimension had a strong correlation with the PWI global item of life satisfaction (*ρ* = 0.61, *P* < 0.001), PWI (*ρ* = 0.58, *P* < 0.001), PWI domains of achievement (*ρ* = 0.53, *P* < 0.001, hypothesis 15), standard of living (*ρ* = 0.49, *P* < 0.001, hypothesis 16) and future security (*ρ* = 0.48, *P* < 0.001, hypothesis 17) (Table 6).

### Pain management

The dimension of pain management demonstrated a moderate and significant correlation with the EQ-5D-5L (*ρ* = 0.41, *P* < 0.001) and the PWI domain of health (Table 6). However, it did not show high correlation with the PWI domain of personal health (*ρ* = 0.35, *P* < 0.001, hypothesis 18) and EQ-5D VAS (*ρ* = 0.37, *P* < 0.001, hypothesis 19) as expected.

### Activity

The activity dimension demonstrated a stronger correlation with the PWI (*ρ* = 0.54, *P* < 0.001) and the PWI domain of achievements (*ρ* = 0.53, *P* < 0.001, hypothesis 20). It demonstrated moderate correlation with the ASCOT domains for occupation (*ρ* = 0.45, *P* < 0.001, hypothesis 21) (Table 6).

Of the 21 priori hypotheses constructed (Table 2), 18 hypotheses (85.7%) were met suggesting that the QOL-ACC descriptive system and its dimensions have demonstrated adequate evidence of convergent validity.

### Known-group validity

**Fig. 1:**
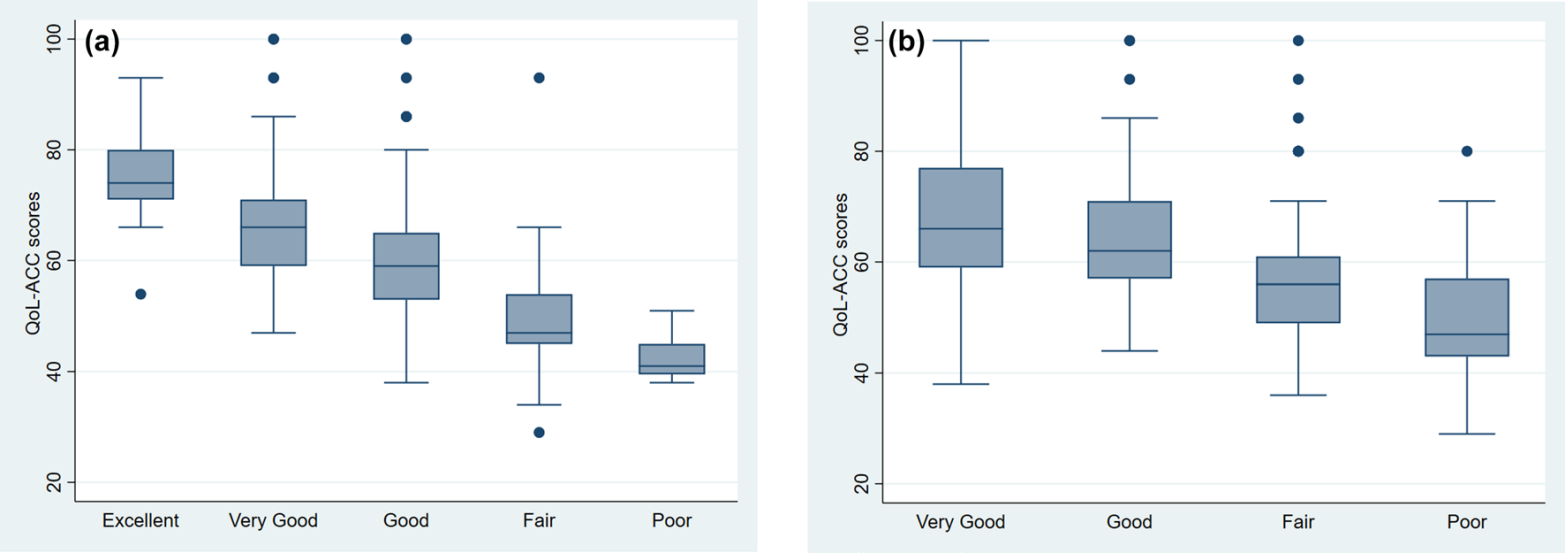
**a** The Quality-of-Life-Aged Care Consumers (QOL-ACC) scores by self-rated quality of life**. b** The Quality-of-Life-Aged Care Consumers (QOL-ACC) scores by self-rated health

**Fig. 2:**
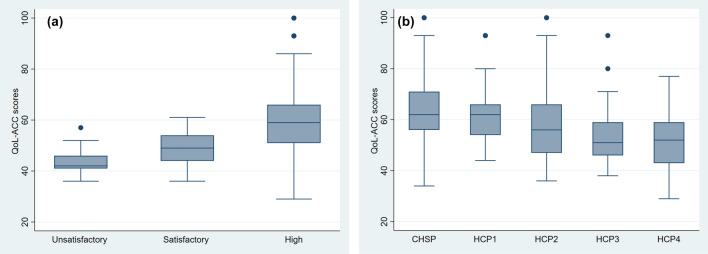
**a** The Quality-of-Life-Aged Care Consumers (QOL-ACC) scores by quality of aged care service experience measured by the Quality of Care Experience-Aged Care Consumers (QCE-ACC). **b** The Quality-of-Life-Aged Care Consumers (QOL-ACC) scores by different types of community-aged care service types (*CHSP* Commonwealth Home Support Programme, *HCP* Home Care Package)

The QOL-ACC discriminated across respondents with different self-reported QOL ratings (Fig. 1a), health ratings (Fig. 1b), aged care quality experience categories (Fig. 2a) and those receiving different levels of community-aged care services (Fig. 2b).

Self-reported QOL ratings were associated with poor QOL-ACC scores (Chi-squared = 121.4, df = 4. *P* < 0.001) with significant differences between groups. Self-reported health ratings were also associated with poorer QOL-ACC scores (Chi-squared = 69.1, df = 3, *P* < 0.001) with significant differences within and between all groups except between very good and good ratings (*P* = 0.10).

Higher aged care quality experience was associated with higher QOL-ACC scores (Chi-squared = 33.1, df = 2, *P* < 0.001). There was statistically significant difference in QOL-ACC scores between those who had high versus satisfactory (*P* < 0.001) and high versus unacceptable (*P* < 0.001) aged care quality experience but not between individuals who had unacceptable versus satisfactory (*P* = 0.46) aged care quality experience. Individuals who were receiving higher levels of care and support (HCP3&4) had lower QOL-ACC scores on average than those receiving lower levels of care and support (CHSP, HCP1&2) (Chi-squared = 30.3, df = 4, *P* < 0.001).

## Discussion

This study has provided evidence of the construct validity of the QOL-ACC descriptive system both at the overall and dimension level in a sample of older people receiving community-aged care services. The current study builds upon our recent studies reporting on content (dimension and items) generation and refinement with the direct involvement of older people receiving aged care services and aged care industry representatives ensuring the practicality, face and content validity of the QOL-ACC [12, 14, 15]. Our analyses showed that the QOL-ACC descriptive system demonstrated expected correlations with similar constructs, and known-group validity, differentiating respondents by different levels of self-reported health, QOL, care quality and care needs. The final stage of development for the QOL-ACC is a valuation study which will develop an accompanying preference-based scoring algorithm based upon preferences of a large sample of older people receiving aged care services and provide further evidence of construct validity in both home and residential care settings.

The QOL-ACC demonstrated positive and significant correlations with the PWI, ASCOT and EQ-5D-5L. Our analyses suggested strong correlations but not strong enough to indicate that the QOL-ACC and the constructs measured by other instruments were identical or redundant. As hypothesised, the relationship between the QOL-ACC and PWI was stronger than with the ASCOT and EQ-5D-5L. The QOL-ACC also showed a stronger relationship with the PWI item of global life satisfaction and domain of achievement. These findings are consistent with other studies suggesting that life satisfaction is intrinsic to a better QOL perception in older people and older people who are more satisfied with the care and support they are receiving are more likely to engage with aged care services leading to better outcomes [36, 37]. The QOL-ACC demonstrated a slightly stronger correlation with ASCOT than with the EQ-5D-5L. This finding is unsurprising as the QOL-ACC has been designed primarily for application with older people to assess their QOL in an aged care context rather than a health system context. As such in common with the ASCOT it captures wider aspects of QOL than the EQ-5D which has a narrower focus on health-related QOL.

The QOL-ACC also demonstrated a moderate but positive correlation with the QCE-ACC (a measure of quality of care experience). The strength of correlations between these two instruments was modest confirming that good quality of care impacts positively on older people QOL, although suggesting that the constructs of QOL and quality of care experience provide sufficiently different information in the aged care context. The finding is not surprising as the QCE-ACC was designed to measure care experience and largely reflective of care processes rather than QOL and/or well-being outcomes. Application of these two short instruments across the aged care sector would facilitate identification of the characteristics of aged care and the care environment that contribute positively towards better care experience and QOL outcomes from the users’ perspectives.

This study has also provided evidence of the convergent validity of the QOL-ACC with important constructs of QOL that matters to older people. Social connection and social support have been identified as a significant component and modifier of QOL in older people [38]. Strong and positive relationships with family and friends are significant particularly for older people who may be dealing with stressful events including health shocks, declines in health over time and having to rely on others for care and support. By demonstrating a strong relationship with the ASCOT domain of social participation and PWI domains of community and relationships (Table 5), our data suggested that the QOL-ACC sufficiently captures the social component of the QOL. The QOL-ACC also captures important constructs related to emotional well-being and independence by demonstrating its strong relationships with the ASCOT domain of control, PWI domains of future security and achievements. Another significant QOL enhancing characteristic for older people is their ability to engage in activities that matter to them [12]. The ability of the QOL-ACC to capture the construct of activity was demonstrated by its association with the ASCOT domain of occupation.

As hypothesised the QOL-ACC dimension of pain management had a moderate and significant correlation with the EQ-5D-5L and EQ-VAS, but mostly very weak or no relationship with other instruments and their domains. The strength of the relationship between the QOL-ACC dimension of pain management and EQ-5D-5L which has a pain dimension was significant but modest. The inclusion of a pain management dimension over “pain perception” dimension in the QOL-ACC descriptive system was a conscious decision (also supported by the qualitative data). Older people frequently commented that aches and pains are an expected part of ageing and that QOL was significantly impacted by the extent to which their pain is managed well [39]. The QOL-ACC dimension of emotional well-being demonstrated a strong relationship with the PWI and PWI global item of life satisfaction, indicating an intrinsic relationship between mental health and perceived life satisfaction.

The QOL-ACC was able to discriminate between older people with different health and QOL ratings, i.e. respondents with better self-reported health and QOL were associated with better scores and vice versa, signifying its known-group validity. The QOL-ACC also discriminated between those receiving different levels of community-aged care services with increasing care levels (a proxy measure of higher care needs) associated with lower QOL-ACC scores, indicating poorer quality of life overall. However, there was no statistical difference in total QOL-ACC scores for older people receiving two adjacent level of home care services (e.g. CHSP vs HCP1, HCP2 vs HCP3 and HCP 3 vs HCP 4). This may reflect that older people receiving adjacent level of care services are relatively more homogenous in terms of their care needs than those at wider home care level intervals (e.g. HCP1 vs HCP3). In addition, many older Australians currently receiving lower-level HCP have been approved for a higher-level HCP but remain on a national waiting list on lower-level packages due to a shortage with current waiting times of up to 21 months to receive approved level of HCPs [40].

There are several limitations to this study that are important to highlight. Firstly, due to COVID-19 restrictions during the period of data collection, we were unable to facilitate data collection in harder to reach groups including older people who do not have access/are not familiar with computers and surveys administered via the internet. We were also not able to include older people from culturally and linguistically diverse groups (CALD) unable to read English because of resource limitations resulting in inability to offer translated surveys in multiple languages. In addition, whilst being more consistent with the pattern of national representation of the provision of aged care services in the community (Table 3) older people receiving higher-level (levels 3 and 4) home care packages were under-represented relative to older people receiving lower-level home care packages (levels 1 and 2) and those receiving community aged care services (Table 3).

In conclusion, this study provides strong evidence for the construct validity of the QOL-ACC descriptive system and its six dimensions to assess aged care-specific quality of life among older Australians using community and home-based aged care services in Australia. The QOL-ACC has been co-designed from its inception with older people accessing aged care services ensuring its high content validity. We are currently developing a preference-weighted value set to accompany the QOL-ACC descriptive system, specific to older people using aged care services, which will be subjected to a series of rigorous validity and reliability assessments. We envision that the QOL-ACC will be utilised to incorporate quality of life as a new quality indicator for aged care and also as a tool for economic evaluation of new service models and technologies in aged care. Routine measurement and public reporting of QOL across service providers would provide important information for aged care consumers and also for individual providers to benchmark their service quality against national standards, facilitating interventions to improve QOL leading to improved service quality and QOL outcomes for older people.[41]

## Plain English summary

With a rapid increase in life expectancy and the aging of the population, more and more older people in Australia and internationally are accessing aged care services at some point in their lives to obtain care and support either in their own homes or in a residential care facility. Quality of life is an important measure to capture aged care users’ perspectives on their own lives, therefore, can be integrated as a part of a routine assessment of care quality in aged care sector. This study investigates the construct validity of the Quality-Of-Life-Aged Care Consumers (QOL-ACC), a new quality-of-life tool co-created from inception with older people accessing aged care services in home and residential care settings. The findings from this study demonstrate the unique properties of the QOL-ACC as a robust and valid measure of quality of life for older people accessing home and community based aged care services.

## Supplementary Information

Below is the link to the electronic supplementary material.

Supplementary file1 (DOC 298 kb)

## Data Availability

Not applicable.
