# Supplementary material for: Assessing the construct validity of the Quality-of-Life-Aged Care Consumers (QOL-ACC): an aged care-specific quality-of-life measure
Source: Qual Life Res. 2022 Jun 9;31(9):2849–65. doi: 10.1007/s11136-022-03142-x (PMC9181894; doi:10.1007/s11136-022-03142-x)
Supplement: Supplementary file 1 — Supplementary file1 (DOC 298 kb) [file 11136_2022_3142_MOESM1_ESM.doc]

**Appendix S1: Assessing the distributions of the QOL-ACC, ASCOT, QCE-ACC and PWI scores**

**Aim:** To assess if the QOL-ACC, ASCOT, QCE-ACC and PWI scores were normally distributed to inform type of statistical tests appropriate to assess the construct validity the QOL-ACC.

**Methods:** A series of histograms were plotted for each instrument’s scores and the distribution of the scores were visually inspected with a plotted normal distribution curve. If the scores are normally distributed, then parametric tests will be used, otherwise non-parametric tests will be used.

**Results:** All histograms are shown in Figure S2.1. The QOL-ACC (Figure S2.1A) scores demonstrated a normal distribution, however the scores of other instruments did not fit a normal distribution. The scores of the EQ-5D-5L (Figure S2.1B), PWI (Figure S2.1C), ASCOT (Figure S2.1D), QCE-ACC (Figure S2.1E) and EQ VAS (Figure S2.1F) were left skewed suggesting that most of the respondents had better scores.

**Conclusion:** The QOL-ACC scores showed a normal distribution however the scores from other instruments did not fit to a normal distribution. Therefore, non-parametric tests were used.

**Figure S1.1:** Histograms showing the distribution of the scores of QOL-ACC (S2.1A), EQ-5D-5L (S2.1B), PWI (S2.1C), ASCOT (S2.1D), QCE-ACC (S2.1E) and EQ VAS (S2.1F). *Note: QOL-ACC= Quality of life- Aged Care Consumers, EQ-5D-5L= EuroQOL 5 dimension and 5 level, PWI= Personal Well-being Index, ASCOT= Adult Social Care Outcome Tool, QCE-ACC= Quality of Care Experience- Aged Care Consumers, EQ VAS= EuroQOL Visual Analogue Scale.*

**
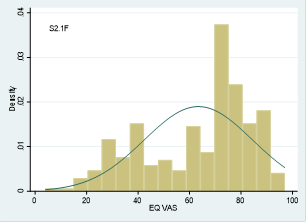
**

**Appendix S2: Assessing convergent validity of the Quality of Life- Aged Care Consumer (QOL-ACC) instrument against other instruments using locally weighted scatterplot (LOWLESS)**

**Aim:** To assess the convergent validity of the QOL-ACC against preference-based measures (EQ-5D-5L, and ASCOT), quality of care experience (QCE-ACC), measure of life satisfaction (PWI), global item of health (EQ-VAS) and global item of life satisfaction (PWI-global item of life satisfaction).

**Methods:** Locally weighted scatterplot smoothing (LOWESS) is a non-parametric regression method which plots a line of central tendency in a scatter plot of two instruments and it demonstrates a general relationship across the possible scale ranges without making assumption about the actual relationships. LOWESS plots were used to visually assess strength and direction of the relationship between the QOL-ACC and other constructs (instruments). These plots complimented the correlations statistics described in the manuscript.

**Results:** Figure S3.1 presents the LOWESS plots between the QOL-ACC and other constructs. The upward trends in relationship between the QOL-ACC and other instruments indicated that better QOL scores measured by the QOL-ACC was associated with higher (better) scores in other instruments.[1] The trends was much pronounced between the QOL-ACC and the PWI/ASCOT/EQ-5D-5L (Figure S3.1) than with the QOL-ACC and the QCE-ACC, emphasising the strength of correlations depicted in Table 6. Similarly, the upward trend was much pronounced between the QOL-ACC and PWI global item of life satisfaction than with the EQ-VAS across the full range of the scores, again mirroring the strength of correlation depicted in Table 6.

**Conclusion:** The LOWESS plots complimented the correlation results described in the manuscript, adding further evidence to prove convergent validity of the QOL-ACC with other instruments.

**Figure S.1:** The QOL-ACC LOWESS plots (clockwise) againsts the EQ-5D-5L (S3.1A), EQ-VAS (S3.1B), QCE-ACC (S3.1C), ASCOT (S3.1D), PWI (S3.1E) and PWI global item of life satisfaction (S3.1F). *Note: QOL-ACC= Quality of life- Aged Care Consumers, EQ-5D-5L= EuroQOL 5 dimension and 5 level, PWI= Personal Well-being Index, ASCOT= Adult Social Care Outcome Tool, QCE-ACC= Quality of Care Experience- Aged Care Consumers, EQ VAS= EuroQOL Visual Analogue Scale.*

**Appendix Figure S3. The QOL-ACC scores (median and IQR) stratified by self-reported quality of life, health, aged care quality standard and types of community aged care services.**

**Reference**

1. Cleveland, W. S. (1979). Robust locally weighted regression and smoothing scatterplots. J Am Stat Assco, 74(368), 829-836.
